# Rehabilitation Needs, Service Provision, and Costs in the First Year Following Traumatic Injuries: Protocol for a Prospective Cohort Study

**DOI:** 10.2196/25980

**Published:** 2021-04-14

**Authors:** Helene Lundgaard Soberg, Håkon Øgreid Moksnes, Audny Anke, Olav Røise, Cecilie Røe, Eline Aas, Unni Sveen, Christine Gaarder, Pål Aksel Næss, Eirik Helseth, Hilde Margrete Dahl, Frank Becker, Marianne Løvstad, Kristian Bartnes, Christoph Schäfer, Mari S Rasmussen, Paul Perrin, Juan Lu, Torgeir Hellstrøm, Nada Andelic

**Affiliations:** 1 Department of Physical Medicine and Rehabilitation Oslo University Hospital Oslo Norway; 2 Faculty of Health Sciences Oslo Metropolitan University Oslo Norway; 3 Institute of Health and Society, Research Centre for Habilitation and Rehabilitation Models & Services (CHARM) Faculty of Medicine University of Oslo Oslo Norway; 4 Department of Rehabilitation University Hospital of North Norway Tromsø Norway; 5 Faculty of Health Sciences Department of Clinical Medicine University of Tromsø, The Arctic University of Norway Tromsø Norway; 6 Division of Orthopaedic Surgery Oslo University Hospital Oslo Norway; 7 Institute for Clinical Medicine Faculty of Medicine University of Oslo Oslo Norway; 8 Department of Health Management and Health Economics Institute for Health and Society University of Oslo Oslo Norway; 9 Department of Traumatology Oslo University Hospital Oslo Norway; 10 Department of Neurosurgery Oslo University Hospital Oslo Norway; 11 Department of Child Neurology Oslo University Hospital Oslo Norway; 12 Sunnaas Rehabilitation Hospital Nesoddtangen Norway; 13 Department of Psychology Faculty of Social Sciences University of Oslo Oslo Norway; 14 Department of Cardiothoracic and Vascular Surgery University Hospital of North Norway Tromsø Norway; 15 Departments of Psychology and Physical Medicine and Rehabilitation Virginia Commonwealth University Richmond, VA United States; 16 Division of Epidemiology Department of Family Medicine and Population Health Virginia Commonwealth University Richmond, VA United States

**Keywords:** trauma, rehabilitation needs, health services, costs, rehabilitation, traumatic injury, injury

## Abstract

**Background:**

Traumatic injuries, defined as physical injuries with sudden onset, are a major public health problem worldwide. There is a paucity of knowledge regarding rehabilitation needs and service provision for patients with moderate and major trauma, even if rehabilitation research on a spectrum of specific injuries is available.

**Objective:**

This study aims to describe the prevalence of rehabilitation needs, the provided services, and functional outcomes across all age groups, levels of injury severity, and geographical regions in the first year after trauma. Direct and indirect costs of rehabilitation provision will also be assessed. The overarching aim is to better understand where to target future efforts.

**Methods:**

This is a population-based prospective follow-up study. It encompasses patients of all ages with moderate and severe acute traumatic injury (New Injury Severity Score >9) admitted to the regional trauma centers in southeastern and northern Norway over a 1-year period (2020). Sociodemographic and injury data will be collected. Upon hospital discharge, rehabilitation physicians estimate rehabilitation needs. Rehabilitation needs are assessed by the Rehabilitation Complexity Scale Extended–Trauma (RCS E–Trauma; specialized inpatient rehabilitation), Needs and Provision Complexity Scale (NPCS; community-based rehabilitation and health care service delivery), and Family Needs Questionnaire–Pediatric Version (FNQ-P). Patients, family caregivers, or both will complete questionnaires at 6- and 12-month follow-ups, which are supplemented by telephone interviews. Data on functioning and disability, mental health, health-related quality of life measured by the EuroQol Questionnaire (EQ-5D), and needs and provision of rehabilitation and health care services are collected by validated outcome measures. Unmet needs are represented by the discrepancies between the estimates of the RCS E–Trauma and NPCS at the time of a patient’s discharge and the rehabilitation services the patient has actually received. Formal service provision (including admission to inpatient- or outpatient-based rehabilitation), informal care, and associated costs will be collected.

**Results:**

The project was funded in December 2018 and approved by the Regional Committee for Medical and Health Research Ethics in October 2019. Inclusion of patients began at Oslo University Hospital on January 1, 2020, and at the University Hospital of North Norway on February 1, 2020. As of February 2021, we have enrolled 612 patients, and for 286 patients the 6-month follow-up has been completed. Papers will be drafted for publication throughout 2021 and 2022.

**Conclusions:**

This study will improve our understanding of existing service provision, the gaps between needs and services, and the associated costs for treating patients with moderate and major trauma. This may guide the improvement of rehabilitation and health care resource planning and allocation.

**International Registered Report Identifier (IRRID):**

DERR1-10.2196/25980

## Introduction

Traumatic injuries, defined as physical injuries with sudden onset and a severity level requiring immediate medical attention, are a major public health problem worldwide [1]. The most common injuries occur in the extremities (38.3%), head/brain (35.4%), thorax (29.5%), and spine (24.3%) [2]. These injuries are the leading cause of problems in physical, cognitive, emotional, behavioral, and psychosocial functioning, interfering with daily life, work, and health-related quality of life (HRQL) [3-6]. Hemorrhage from internal organs usually does not cause major long-term disabilities. Still, hemorrhage is an important contributor to early deaths and serious complications after trauma including systemic inflammatory response syndrome, multi-organ failure, and acute respiratory distress syndrome [7]. Survivors of these injuries are patients who are catabolic over time, with massive muscle loss, and will have high rehabilitation needs.

While acute trauma care is considered to be of high quality in Norway, rehabilitation services remain fragmented [8,9]. Gabbe et al [4] reported that 80% of major trauma patients suffered problems affecting their daily life 6 months post injury, whereas Soberg et al [5] found that at 2 years post injury, self-reported health and functioning and work participation were significantly lower than in the general population [10,11].

Pediatric trauma constitutes an important subset of traumatic injuries, with lower mortality rates than injuries in adults but a high risk of lifelong functional impairment and disability [12]. Gabbe et al [12] reported that severely injured children showed ongoing disability and reduced HRQL 12 months after injury. Younger children with severe traumatic brain injury (TBI) are at the highest risk of long-term behavioral and learning disabilities and of having unmet or unrecognized health care needs during the first year after injury [13-15].

Trauma rehabilitation should be provided as a set of coordinated, multidisciplinary services tailored to the patient’s needs. Personal factors such as resilience, personality, chronic pain, and access to social support are powerful predictors of outcome [16]; however, the severity of the injury is not a potent predictor of psychological outcomes [17,18]. Thus, these are factors that can affect individuals’ perceived rehabilitation needs and should be taken into account in research on the provision of rehabilitation services.

“A need for rehabilitation” refers to any needs that can be met with rehabilitation management, interventions, and services in the acute, subacute, and postacute phases of an injury. Such needs relate both to specialized inpatient and outpatient services and those provided in community-based settings [19]. A discrepancy between general rehabilitation provision and the need for rehabilitation services has been documented internationally [19]. The extent of service provision may vary across different municipalities and across regions, yet no study has been conducted in Norway to explore how geographical differences in rehabilitation services influence rehabilitation pathways, functioning, and health outcomes of patients with moderate and major trauma [20]. Internationally, research diverges regarding regional differences between high- and low-density population areas. In a Canadian study on multiple trauma survivors, no regional differences were found, but numerous barriers to rehabilitation services were reported [21]. In a survey of rehabilitation services in Australia, Graham and Cameron [22] found that there was better access to rehabilitation services in metropolitan areas. Given the paucity of data on the impact of geography on postinjury outcomes, there is a need for a comprehensive evidence base on regional variation in recovery and rehabilitation to optimize postacute services [23].

Few studies exist on the cost-effectiveness of rehabilitation services after traumatic injuries [24]. Patients with complex disabilities after trauma are costly to treat; however, rehabilitation is considered a worthwhile societal investment in the trauma survivors’ regaining of independence and HRQL [25,26]. By applying the Needs and Provision Complexity Scale (NPCS) and a cost assessment algorithm, a provision shortfall of community-based rehabilitation services that may also contribute to increased care burden and costs for family caregivers was reported [27,28] Studies from other countries are needed to confirm these findings. Furthermore, to address shortfalls in service provision to trauma patients, longitudinal, population-based data are required. It can also indicate a baseline for evaluating future system-level changes and service development.

The main aims of this study are to explore the rehabilitation needs and provided services for patients after moderate and major trauma and to study changes in needs over the first year. The study will evaluate the implementation of recommendations on early rehabilitation and continuity of care, as stated in the National Trauma Plan in Norway [8], and assess the specialized and community-based rehabilitation provision. The connections between patients’ rehabilitation needs and functional levels will be explored, and geographical variation in service provision, patient outcomes, and the associated costs will be studied. The main hypotheses are as follows: 1) Underprovision of coordinated rehabilitation services and continuity of care will lead to unmet rehabilitation needs in the trauma patients regardless of type of trauma, demography, postinjury physical and psychological functioning, or regional affiliation. 2) There is geographical variation in patient outcomes for severely injured patients. 3) Rehabilitation is cost-effective; however, there are large societal costs from informal care provided by caregivers.

## Methods

### Design

This is a multicenter, population-based study with prospective follow-ups at 6 and 12 months post injury.

### Study Settings

The regional trauma centers in the southeastern and northern parts of Norway are Oslo University Hospital (OUS) and the University Hospital of North Norway (UNN), respectively. We classify the northern region as more rural due to the long distances to and from hospital and rehabilitation facilities and the low population density. The southeastern region is classified as mainly urban with shorter distances to and from hospital and rehabilitation facilities and higher population density. The prehospital emergency medical services are well organized in both regions [29].

### Participants

The group eligible for the study will be approximately 600 patients of all ages with moderate and major trauma, including multiple injuries (extremities, head/brain, thorax, spine, abdomen, face, neck, external/other), who were consecutively admitted to the regional trauma centers in the southeastern and northern parts of Norway over a 1-year period and subsequently discharged.

### Inclusion and Exclusion Criteria

All Norwegian residents may be included who were admitted directly to the above hospitals or admitted after transfer from local hospitals within 72 hours after injury, with at least a two-day hospital stay and a New Injury Severity Score (NISS) greater than 9 (ie, moderate to severe injuries). Non-Norwegian residents and deceased patients will be excluded.

### Inclusion Procedure

All patients admitted with a trauma alarm will be assessed daily by the participating doctor in the project with formal expertise in scoring the severity of the injuries. Once it is clarified that a patient meets the inclusion criteria, the patient is informed by the research assistant, and informed consent is obtained.

### Ethical Considerations, Procedure, and Data Collection

All components of the research project will be conducted according to the ethical guidelines of the Declaration of Helsinki and approved by the Regional Committee for Medical and Health Research Ethics (approval number 31676) and the Norwegian Data Inspectorate (approval number 19/26515). Data will be handled and stored securely on the research servers at OUS and UNN (patient-related data) and the Service for Sensitive Data at the University of Oslo (cost data). All data will be deidentified when sharing them with partners and in analysis and presentations.

Regional research assistants and PhD students will oversee patient recruitment and data collection. The patients will be identified during acute admission to hospital. Trauma patients with NISS>9 are included in both hospitals’ trauma registries and the National Trauma Registry (NTR). It will thus be possible to validate the trauma severity scores of included patients as well as attrition rates in this study. These scores derive from the Abbreviated Injury Scale (AIS), Injury Severity Score (ISS), and NISS.

Information about the study will be presented to patients in written and oral form with informed consent emphasizing their right to withdraw from the project at any time without requiring a reason. The recruited patients (or their proxy or caregiver) must provide this informed consent. For pediatric patients, the information will be given in written and oral form, and forms adapted to the language of the youngest children will be provided. For children and adolescents younger than 18 years, parents must give written consent. Adolescents aged 16-17 years must sign consent for themselves as well. For adult patients with cognitive or communication difficulties, a family member or caregiver will be identified to provide consent (ie, deputy consent) and assist in completing the questionnaires.

### Baseline Data Collection and Registration

Collection and registration will be based on information from medical records. The trauma scores will be validated by data registered by certified AIS registrars in the hospitals’ trauma registries and, if necessary, in connection with other available clinical information about the patients’ stays in hospital departments.

### Patient Characteristics

Sociodemographic data will include family status (marital status and children), preexisting comorbidity, education or work status, and substance use at the time of injury. Injury-related data include diagnoses (International Statistical Classification of Diseases, Tenth Revision diagnosis codes of S00-T32); cause and type of injury; severity of injury as assessed by the NISS, ISS, and AIS; nonsurgical and surgical treatments; treatment complications; time spent on a ventilator; length of hospital stay; and discharge location.

### Injury Severity

The NISS is used to define injury severity in the inclusion phase (scores 9-15 are moderate, 16-24 are severe, and 25-75 are profound). The rationale for the inclusion criterion of an NISS >9 is based on guidance from the National Institute for Health and Care Excellence that such patients should be assessed for rehabilitation needs, and a rehabilitation prescription should be provided for all patients who are deemed to have those rehabilitation needs [30].

### Primary Outcomes: Rehabilitation Needs

The Rehabilitation Complexity Scale Extended–Trauma (RCS E–Trauma) is used for identification of needs for specialized inpatient rehabilitation. The RCS E–Trauma is a simple 5-item scale (score range 0-25) that reflects rehabilitation resource requirement for medical, basic care and support, skilled nursing, and equipment needs and therapy inputs [31]. The needs for specialized primary in-hospital rehabilitation (ie, rehabilitation commencing immediately after acute treatment) will be estimated in accordance with clinical judgement by a doctor specialized in rehabilitation medicine at discharge from acute care at the trauma hospital. After 6 months, a medical doctor will use information from a telephone interview with the patient, the caregiver, or both and the medical records to evaluate whether the estimated needs for primary in-hospital rehabilitation were met or not.

The NPCS in its clinician version evaluates needs for community-based rehabilitation and health care service delivery [27]. The NPCS is a pragmatic instrument for measuring both an individual’s needs for rehabilitation and support (“NPCS-Needs”) and the levels of service provided (“NPCS-Gets”) within a given period. An algorithm has been developed to express the impact of met and unmet needs in terms of costs. The NPCS is a 15-item measure that consists of two parts with six subscales and a total score range of 0-50. Part A (NPCS-Needs) is completed by one or more clinicians to evaluate each patient’s needs for health and social care in a given period. Part B (NPCS-Gets) is a mirror image of the same tool, completed at the end of that period, to evaluate the levels of service that have been provided in relation to those needs. Further, the NPCS consists of two main domains. The first is “Health and personal care needs” (score range 0-25), which includes the following subscales: “Health care” (score range 0-6), “Personal care” (score range 0–10), and “Rehabilitation” (score range 0-9). The second is “Social care and support needs” (score range 0-25), which includes the following subscales: “Social and family support” (score range 0-13), “Equipment” (score range 0-3), and “Environment” (score range 0-9) [27]. The expected needs for community-based services during the first 6 months will be estimated in accordance with the clinical judgement of a specialist in rehabilitation medicine upon discharge from the trauma hospital.

The NPCS also includes a self-report version. Patients or carers are asked to report the level of services received within the last 6 months and whether they consider this to be the right amount, too much, or too little (a free-text box is provided for elaboration). The NPCS patient version will be used at 6- and 12-month intervals as an integral part of the telephone interview discussion, measuring the extent to which the self-perceived needs have been met through service provision and informal care. The 6-month report will be used to estimate needs for community-based rehabilitation and health care service delivery for the 6- to 12-month period post discharge. The NPCS will in this study serve as a base for estimation of costs of services received after discharge from the specialist health service.

The Family Needs Questionnaire–Pediatric Version (FNQ-P) [32] will be used to assess the family needs in children across six categories: “Health information,” “Emotional support,” “Community support,” “Instrumental support,” “Professional support,” and “Involvement with care.” Further, formal service provision, including admission to inpatient-, outpatient-, or community-based rehabilitation (as well as informal care) and associated costs will be collected.

Primary and secondary outcomes (ie, measures of functional outcomes, psychological functions, and HRQL) at 6 and 12 months are presented in Table 1.

**Table 1:** Primary and secondary outcome measures at 6 and 12 months post injury.

Outcomes		Participants	Description	Completed by
**Primary outcomes**				
	Rehabilitation Complexity Scale Extended–Trauma (RCS E–Trauma) [31]	Adult/child	Estimation of specialized inpatient rehabilitation needs	Rehabilitation specialist
	Needs and Provision Complexity Scale (NPCS) A (Needs) and B (Gets) [27]	Adult/child	Estimation of community-based rehabilitation needs and registration of community-based rehabilitation provided (Gets)	Clinician version: rehabilitation specialist Patient version: patients by interview
	Family Needs Questionnaire–Pediatric Version (FNQ-P) [32]	Families of children and youth	A measure for assessing the degree to which the family’s needs have been met	Caregivers for children 2-18 years
**Secondary outcomes**				
	Glasgow Outcome Scale Extended (GOSE) [33]	Adult/child	A global outcome commonly used in trauma research	Clinician
	Resilience Scale for Adults (RSA) [34]	Adult	A measure of resilience	Patients
	Patient Health Questionnaire 9 (PHQ-9) [35]	Adult	A screening of depression	Patients
	Generalized Anxiety Disorder 7 (GAD-7) [36]	Adult	A screening of anxiety	Patients
	Impact of Event Scale–Revised (IES-R) [37]	Adult	A measure of presence of subjective distress in adults	Patients
	Children’s Revised Impact of Event Scale (CRIES-8) [38]	Child/parent	A measure of subjective distress in pediatric cases	Children from 8 years of age; caregivers
	Strengths and Difficulties Questionnaire (SDQ) [39]	Child	A brief behavioral measure (4-17 years)	Caregivers for children 4-17 years Children 11-17 years
	Return to work/school	Adult/family caregiver/ child/parent	Work/school status, return to same role/position, benefits from the labor welfare system at individual and family levels	Clinicians by interview
	WHO^a^ Disability Assessment Schedule 2.0 (WHODAS 2.0) [40]	Adult	A measure of functioning and disability: cognition, mobility, self-care, getting along, life activities and participation	Patients/caregivers
	EuroQol Questionnaire (EQ-5D) [41]	Adult/family caregiver/ child/parent	A generic measure of health status (mobility, self-care, usual activities, pain/discomfort, anxiety/depression); health profiles and a weighted total value for HRQL	Patients/caregivers Parent proxy for children 0-15 years Children: 8-15 years
	Pediatric Quality of Life Inventory (Peds-QL) 4.0 Generic Core Scales [42]	Child/parent	Patient’s and parent’s perceptions of quality of life	Parents: parent proxy for toddlers (2-4 years), young children (5-7 years), children (8-12 years), and teens (13-18 years) Children/teens: self-report for 5-7 years, 8-12 years, and teens (13-18 years)

^a^WHO: World Health Organization.

All measures exist in Norwegian versions. The estimated time taken for the follow-up interviews and questionnaires at 6 and 12 months is approximately 1.5 to 2.5 hours.

### Follow-up Data Collection

Questionnaires for patients, their caregivers, or both will be completed at the 6- and 12-month follow-ups by mail or electronic link supplemented by a telephone interview. This will provide data on changes in sociodemographic characteristics, measures of functioning and disability, mental health, and HRQL, as well as rehabilitation, health and social care service needs, and provision of those services (Gets). The discrepancy between the scores of RCS E–Trauma and NPCS assessed at discharge (Needs) and the rehabilitation services received (Gets) during the first 6 months represents unmet needs. Unmet rehabilitation needs at 12 months are the discrepancy between NPCS-Needs as evaluated by patients at 6 months and NPCS-Gets (receiving services 6-12 months after injury) at 12 months.

### Cost Estimation

We aim to estimate costs of a traumatic injury from a societal perspective, not only as consequences related to medical treatment but also consequences for work and family situations. Health care utilization encompasses in-hospital stays, including rehabilitation and outpatient appointments, as well as home care and contact with community-based rehabilitation services, multidisciplinary rehabilitation, individual rehabilitation plans, and other health care services. Further, use of social support professionals, vocational and educational rehabilitation, equipment and assistive technology, income and accommodation, informal care provided by family and significant others, and other cost-related variables will be registered. In this study, participants will record the type and amount of services received during the previous 6 months after 6 months and 12 months following discharge from the trauma hospital. The NPCS patient version will be used at 6- and 12-month intervals as an integral part of the telephone interview discussion and serves as one of the bases for estimation of the costs of services (within the subscales “Health care,” “Personal care,” “Rehabilitation,” “Social and family support,” “Equipment,” and “Environment”) received after discharge from the specialist health service. Loss of productivity due to sick leave and absence from work because of treatment, rehabilitation, or follow-up will be estimated together with costs for the family (both informal and intangible costs).

Total rehabilitation, health care, social support, and societal costs across the periods 0-6 and 7-12 months post discharge will be estimated by combining service utilization and national unit costs. The replacement cost method that evaluates informal care time will be based on the cost of paid professionals at the study time as a “shadow price” for informal care [28]. Intangible costs cannot be directly measured in monetary form, but effects such as pain, joy, or physical and psychological limitations will be assessed using the patient’s and family caregiver’s quality of life according to EQ-5D (EuroQol Questionnaire), which will provide an indication of the patient’s and family caregiver’s HRQL after trauma.

By using the NPCS, an algorithm can be applied to estimate the cost of meeting unmet needs for the purpose of integrating care planning [27]. The costing algorithm was developed in the United Kingdom by professionals with particular experience in managing long-term neurological disabilities and will be adjusted for the Norwegian context of this study. Cost of medication is not included in this study as medication does not present a well-defined rehabilitation service.

### Power Calculation

According to the estimates from the NTR, 931 and 110 patients with NISS >9 were respectively admitted to the trauma referral hospitals in the southeastern and northern parts of Norway in 2015. The 30-day mortality rate for this group was about 10%. In addition, we expect the dropout rate to be around 30% at the 1-year follow-up, comprising a 20% refusal rate and 10% of patients lost to follow-up. Accounting for mortality and dropouts, this study expects to include approximately 600 participants in the final study analyses. A power analysis was performed for a predictive model with an outcome variable collected at two time points (6 months and 12 months), a predictor variable (for example, with two subgroups such as women and men), and an interaction term between time and the predictor. This type of model would be a common model employed in the proposed study and likely the most power-consuming model given the additional power needed to detect an interaction effect. With 80% power (1–β), and assuming an *r*=0.50 correlation between the two measurements of the outcome variable, the estimated sample size of 600 participants would generate enough power to detect all large-sized, medium-sized, and small-sized effects with Cohen f^2^>0.06.

### Statistics

Descriptive statistics and graphing techniques will be used to describe rehabilitation and health care needs, service use, and functional outcomes over time, as well as to compare these factors across age groups, injury severity levels, and geographical regions. The aggregated data from the NTR will be used to estimate the national prevalence of rehabilitation needs and service use after traumatic injuries, including the total number of trauma patients with moderate and severe injury according to age, gender, and geographical region. Unmet needs for specialized inpatient rehabilitation will be based on the evaluation of RCS E–Trauma compared to received services by the 6-month follow-up. Further, unmet community-based rehabilitation needs will be calculated by subtracting the total NPCS-Gets score from the total NPCS-Needs score.

To establish whether the unmet rehabilitation needs differ across subgroups, longitudinal mixed models will be used wherein the nested variable is time (eg, discrepancies between the estimates of needs at discharge and rehabilitation services the patient reported receiving at 6 and 12 months). The models will enable identification of factors that predict unmet rehabilitation needs and differences in the needs based on key demographic, injury-related, or service-related variables. Predictor variables in these models will be left as continuous wherever possible in order to maximize potential variability in the predictors (eg, age, level of education, injury severity scores). Categorical variables will be dichotomized (eg, employment status, marital status, geographical regions). Injuries can be grouped as follows: TBI, spinal cord injuries, orthopedic injuries including amputations, thorax and abdominal injuries including hemorrhaging.

The mean costs of both formal and informal rehabilitation and health care services used during the first year will be calculated and compared across these variables. In this study we have defined informal rehabilitation as help given by family or friends and quantified by hours per week. Information about this is collected through the telephone interview and the results of the patient version of the NPCS. Costs are typically skewed with heavy right tails due to patients with severe injuries using services to a great extent and incurring high levels of cost; when estimating predictors for total costs, we will apply log-linear and general linear models. Additionally, the NPCS algorithm for calculating the costs of meeting unmet needs will be used for the purpose of integrating care planning.

### Data Linkage

For evaluation of completeness and validity of collected data reflecting hospital admissions and service use during the first year after injury, data linkage will be achieved with the Norwegian Patient Register (containing information on diagnoses, length of hospital stays in somatic and rehabilitation units, and costs according to diagnosis-related groups) and with outpatient consultations in the specialist health service. Additionally, linking will be performed with the registries with costs arising from consultations and other contact with emergency services and various health professionals and medical specialists, and with individual-based health care and rehabilitation use in municipal health care services. Sick leave and other benefits data will be extracted from the Norwegian Labour and Welfare Administration. The applications for extracting these data will be sent to the respective registries.

## Results

The project was funded in December 2018 and approved by the Regional Committee for Medical and Health Research Ethics in October 2019. Inclusion of patients began at OUS on January 1, 2020, and at the UNN, Tromsø, on February 1, 2020. As of February 2021, we have enrolled 612 patients. The 6-month follow-up has been completed for 286 patients. Papers will be drafted for publication throughout 2021 and 2022.

## Discussion

This is the first multiregional study in Norway based on data from the NTR on rehabilitation after trauma. We have not found parallel studies on rehabilitation after moderate and major trauma in an international context. This prospective follow-up project is estimated to collect data from over 600 patients and provide innovative insights into rehabilitation needs and costs, as well as intangible costs for patients and family caregivers and their perception of care provided. The study will establish the national prevalence of rehabilitation needs and costs, highlighting patients at risk of unmet rehabilitation needs. Service provision and unmet needs will be evaluated in lieu of outcomes over a broad array of functional domains, thus providing detailed information regarding specific needs.

The results will be of interest across the whole care chain for trauma patients, as there are strong recommendations to plan and initiate the rehabilitation process early in the acute phase. Further, beyond trauma patients, the results will be of beneficial value since this group is representative of patients with long-term disabilities who are expected to have ongoing needs for rehabilitation services and support in general. The results could inform both clinical decision making at the individual level and population-based service planning and delivery in areas capable of improvements.

The project aligns with several national policy documents and white papers and the National Trauma Plan [8,43,44], all of which favor a major strengthening of rehabilitation care in Norway. All of these documents noted a need for reform in this area. They emphasize the need for high-quality and patient-centered care, attentiveness to users’ voices, collaboration between specialist and community health services, integrated and coherent service provision, and long-term support for patients and their caregivers. The documents also express an imperative for individual plans to secure action chains and networks, so that the service provision coalesces as a coherent whole and transitions between responsible services and organizational levels are achieved. Maintaining a user’s perspective implies that an emphasis must be placed on local and flexible solutions that the user can access in his or her everyday life.

The project will inform the World Health Organization global disability action plan 2014-2021 to strengthen and extend rehabilitation provision [45]. The implementation of results could contribute to reducing the burden of injury and improving the lives of people suffering from the consequences of injuries through the development of increasingly individually targeted service delivery. Therefore, the study may highlight both adherence to, and deviation from, the recommendations of the policy documents.

This study also has some limitations that should be discussed. This is an observational study; thus, the causal inferences will be restricted by the design. However, this longitudinal cohort study has a comprehensive and epidemiological approach, comprising registry data and self-reports from patients and caregivers, and the study design provides a higher external validity than an experimental study. Furthermore, as with any longitudinal study, there will be a risk of dropout. To facilitate study adherence and maintain the lowest possible dropout rates, the research team will be trained regarding the follow-up procedures. Baseline data will be available for all participants, allowing comparisons between those who continue in the study and those who drop out. However, since we include patients who are received by the hospitals with trauma alarms, we will not include all patients with injuries with NISS >9, as some with more moderate injuries are not triaged and met by trauma teams.

In conclusion, this study will provide information about rehabilitation needs and service provision for patients who have sustained moderate and major trauma. New knowledge about met and unmet needs will improve the understanding of the existing service provision, the gaps between needs and services, and the associated costs for treating trauma patients, which could guide improvements of rehabilitation and health care resource planning and allocation.

## Abbreviations

AIS: Abbreviated Injury Scale
EQ-5D: EuroQol Questionnaire
FNQ-P: Family Needs Questionnaire–Pediatric Version
HRQL: health-related quality of life
ISS: Injury Severity Score
NISS: New Injury Severity Score
NPCS: Needs and Provision Complexity Scale
NTR: National Trauma Registry
OUS: Oslo University Hospital
RCS E–Trauma: Rehabilitation Complexity Scale Extended–Trauma
TBI: traumatic brain injury
UNN: University Hospital of North Norway
