# Correction: Rehabilitation Needs, Service Provision, and Costs in the First Year Following Traumatic Injuries: Protocol for a Prospective Cohort Study

**DOI:** 10.2196/37723

**Published:** 2022-03-23

**Authors:** Helene Lundgaard Soberg, Håkon Øgreid Moksnes, Audny Anke, Olav Røise, Cecilie Røe, Eline Aas, Unni Sveen, Christine Gaarder, Pål Aksel Næss, Eirik Helseth, Hilde Margrete Dahl, Frank Becker, Marianne Løvstad, Kristian Bartnes, Christoph Schäfer, Mari S Rasmusssen, Paul Perrin, Juan Lu, Torgeir Hellstrøm, Nada Andelic

**Affiliations:** 1 Department of Physical Medicine and Rehabilitation Oslo University Hospital Oslo Norway; 2 Faculty of Health Sciences Oslo Metropolitan University Oslo Norway; 3 Institute of Health and Society, Research Centre for Habilitation and Rehabilitation Models & Services (CHARM) Faculty of Medicine University of Oslo Oslo Norway; 4 Department of Rehabilitation University Hospital of North Norway Tromsø Norway; 5 Faculty of Health Sciences Department of Clinical Medicine University of Tromsø, The Arctic University of Norway Tromsø Norway; 6 Division of Orthopaedic Surgery Oslo University Hospital Oslo Norway; 7 Institute for Clinical Medicine Faculty of Medicine University of Oslo Oslo Norway; 8 Department of Health Management and Health Economics, Institute of Health and Society Faculty of Medicine University of Oslo Oslo Norway; 9 Department of Traumatology Oslo University Hospital Oslo Norway; 10 Department of Neurosurgery Oslo University Hospital Oslo Norway; 11 Department of Child Neurology Oslo University Hospital Oslo Norway; 12 Sunnaas Rehabilitation Hospital Nesoddtangen Norway; 13 Department of Psychology Faculty of Social Sciences University of Oslo Oslo Norway; 14 Department of Cardiothoracic and Vascular Surgery University Hospital of North Norway Tromsø Norway; 15 Departments of Psychology and Physical Medicine and Rehabilitation Virginia Commonwealth University Richmond, VA United States; 16 Division of Epidemiology Department of Family Medicine and Population Health Virginia Commonwealth University Richmond, VA United States

In “Rehabilitation Needs, Service Provision, and Costs in the First Year Following Traumatic Injuries: Protocol for a Prospective Cohort Study” (JMIR Res Protoc 2021;10(4):e25980), several errors were noted in author affiliations.

1. In the originally published paper, author affiliations 1 ("Department of Physical Medicine and Rehabilitation, Oslo University Hospital HF, Oslo, Norway") and 3 (“Department of Physical Medicine and Rehabilitation, Oslo University Hospital, Oslo, Norway”) were the same. Therefore, “Oslo University Hospital HF” has been corrected to “Oslo University Hospital” in this affiliation for the following authors: Helene Lundgaard Soberg, Mari S Rasmusssen, Torgeir Hellstrøm, Nada Andelic. The remaining affiliations have been renumbered as necessary.

2. For Audny Anke, the originally published author affiliation was:

Department of Cardiothoracic and Vascular Surgery, University Hospital of North Norway, Tromsø, Norway

This has been corrected to the following:

Faculty of Health Sciences, Department of Clinical Medicine, University of Tromsø, The Arctic University of Norway, Tromsø, Norway

The following new affiliation has also been added for this author:

Institute of Health and Society, Research Centre for Habilitation and Rehabilitation Models & Services (CHARM), Faculty of Medicine, University

3. For Eline Aas, the originally published author affiliation was:

Department of Health Management and Health Economics, Institute for Health and Society, University of Oslo, Oslo, Norway

This has been corrected to:

Department of Health Management and Health Economics, Institute of Health and Society, Faculty of Medicine, University of Oslo, Oslo, Norway

4. For Marianne Løvstad, the originally published author affiliation was:

Department of Psychology, University of Oslo, Oslo, Norway

This has been corrected to the following:

Department of Psychology, Faculty of Social Sciences, University of Oslo, Oslo, Norway

5. For Kristian Bartnes, the originally published author affiliation was:

Institute of Clinical Medicine, University of Tromsø, The Arctic University of Norway, Tromsø, Norway

This has been corrected to the following:

Faculty of Health Sciences, Department of Clinical Medicine, University of Tromsø, The Arctic University of Norway, Tromsø, Norway

6. For Christoph Schäfer, the originally published author affiliation was:

Department of Cardiothoracic and Vascular Surgery, University Hospital of North Norway, Tromsø, Norway

This has been corrected to the following:

Faculty of Health Sciences, Department of Clinical Medicine, University of Tromsø, The Arctic University of Norway, Tromsø, Norway

The following new affiliation has also been added for this author:

Department of Physical Medicine and Rehabilitation, Oslo University Hospital, Oslo, Norway

In the originally published paper, the complete listing of authors and affiliations was as follows:

Helene Lundgaard Soberg ^1,2*^, PhD; Håkon Øgreid Moksnes ^3,4*^, MD; Audny Anke ^5,6^, MD, PhD; Olav Røise ^7,8^, MD, PhD; Cecilie Røe ^3,7^, MD, PhD; Eline Aas ^9^, PhD; Unni Sveen ^2,3^, PhD; Christine Gaarder ^7,10^, MD, PhD; Pål Aksel Næss ^7,10^, MD, PhD; Eirik Helseth ^7,11^, MD, PhD; Hilde Margrete Dahl ^7,12^, MD, PhD; Frank Becker ^7,13^, MD, PhD; Marianne Løvstad ^13,14^, PhD; Kristian Bartnes ^6,15^, MD, PhD; Christoph Schäfer ^5,6^, MD; Mari S Rasmussen ^1,4^, MSc; Paul Perrin ^16^, PhD; Juan Lu ^17^, MD, PhD; Torgeir Hellstrøm ^1^, MD, PhD; Nada Andelic ^1,4*^, MD, PhD

^1^Department of Physical Medicine and Rehabilitation, Oslo University Hospital HF, Oslo, Norway

^2^Faculty of Health Sciences, Oslo Metropolitan University, Oslo, Norway

^3^Department of Physical Medicine and Rehabilitation, Oslo University Hospital, Oslo, Norway

^4^Institute of Health and Society, Research Centre for Habilitation and Rehabilitation Models & Services (CHARM), Faculty of Medicine, University of Oslo, Oslo, Norway

^5^Department of Rehabilitation, University Hospital of North Norway, Tromsø, Norway

^6^Department of Cardiothoracic and Vascular Surgery, University Hospital of North Norway, Tromsø, Norway

^7^Institute for Clinical Medicine, Faculty of Medicine, University of Oslo, Oslo, Norway

^8^Division of Orthopaedic Surgery, Oslo University Hospital, Oslo, Norway

^9^Department of Health Management and Health Economics, Institute for Health and Society, University of Oslo, Oslo, Norway

^10^Department of Traumatology, Oslo University Hospital, Oslo, Norway

^11^Department of Neurosurgery, Oslo University Hospital, Oslo, Norway

^12^Department of Child Neurology, Oslo University Hospital, Oslo, Norway

^13^Sunnaas Rehabilitation Hospital, Nesoddtangen, Norway

^14^Department of Psychology, University of Oslo, Oslo, Norway

^15^Institute of Clinical Medicine, University of Tromsø, The Arctic University of Norway, Tromsø, Norway

^16^Departments of Psychology and Physical Medicine and Rehabilitation, Virginia Commonwealth University, Richmond, VA, United States

^17^Division of Epidemiology, Department of Family Medicine and Population Health, Virginia Commonwealth University, Richmond, VA, United States

*these authors contributed equally

Corresponding Author:

Helene Lundgaard Soberg, PhD

Department of Physical Medicine and Rehabilitation

Oslo University Hospital HF

PO Box 4956 Nydalen

Oslo, N-0424

Norway

Phone: 47 951009637

Email: h.l.soberg@medisin.uio.no

The complete corrected list of authors and affiliations is as follows:

Helene Lundgaard Soberg ^1,2*^, PhD; Håkon Øgreid Moksnes ^1,3*^, MD; Audny Anke ^3,4,5^, MD, PhD; Olav Røise ^6,7^, MD, PhD; Cecilie Røe ^1,7^, MD, PhD; Eline Aas ^8^, PhD; Unni Sveen ^1,2^, PhD; Christine Gaarder ^7,9^, MD, PhD; Pål Aksel Næss ^7,9^,MD, PhD; Eirik Helseth ^7,10^ MD, PhD; Hilde Margrete Dahl ^7,11^, MD, PhD; Frank Becker ^7.12^, MD, PhD; Marianne Løvstad ^12,13^, PhD; Kristian Bartnes ^5,14^, MD, PhD; Christoph Schäfer ^1,4,5,^ MD; Mari S Rasmussen ^1,3^, MSc; Paul Perrin ^15^, PhD; Juan Lu ^16^, MD, PhD; Torgeir Hellstrøm ^1^, MD, PhD; Nada Andelic ^1,3*^, MD, PhD

^1^Department of Physical Medicine and Rehabilitation, Oslo University Hospital, Oslo, Norway

^2^Faculty of Health Sciences, Oslo Metropolitan University, Oslo, Norway

^3^Institute of Health and Society, Research Centre for Habilitation and Rehabilitation Models & Services (CHARM), Faculty of Medicine, University of Oslo, Oslo, Norway

^4^Department of Rehabilitation, University Hospital of North Norway, Tromsø, Norway

^5^Faculty of Health Sciences, Department of Clinical Medicine, University of Tromsø, The Arctic University of Norway, Tromsø, Norway

^6^Division of Orthopaedic Surgery, Oslo University Hospital, Oslo, Norway

^7^Institute for Clinical Medicine, Faculty of Medicine, University of Oslo, Oslo, Norway

^8^Department of Health Management and Health Economics, Institute of Health and Society, Faculty of Medicine, University of Oslo, Oslo, Norway

^9^Department of Traumatology, Oslo University Hospital, Oslo, Norway

^10^Department of Neurosurgery, Oslo University Hospital, Oslo, Norway

^11^Department of Child Neurology, Oslo University Hospital, Oslo, Norway

^12^Sunnaas Rehabilitation Hospital, Nesoddtangen, Norway

^13^Department of Psychology, Faculty of Social Sciences, University of Oslo, Oslo, Norway

^14^Department of Cardiothoracic and Vascular Surgery, University Hospital of North Norway, Tromsø, Norway

^15^Departments of Psychology and Physical Medicine and Rehabilitation, Virginia Commonwealth University, Richmond, VA, United States

^16^Division of Epidemiology, Department of Family Medicine and Population Health, Virginia Commonwealth University, Richmond, VA, United States

*these authors contributed equally

Corresponding Author:

Helene Lundgaard Soberg, PhD

Department of Physical Medicine and Rehabilitation

Oslo University Hospital

PO Box 4956 Nydalen

Oslo, N-0424

Norway

Phone: 47 951009637

Email: h.l.soberg@medisin.uio.no

The correction will appear in the online version of the paper on the JMIR Publications website on March 23, 2022, together with the publication of this correction notice. Because this was made after submission to PubMed, PubMed Central, and other full-text repositories, the corrected article has also been resubmitted to those repositories.

